# Editorial: Feelings of energy and fatigue: Two different moods

**DOI:** 10.3389/fpsyg.2023.1180285

**Published:** 2023-04-19

**Authors:** Ali Boolani, Joel Martin, Fulvio D'Acquisto, Costantino Balestra

**Affiliations:** ^1^Honors Department, Clarkson University, Potsdam, NY, United States; ^2^Sports Medicine Assessment Research and Testing (SMART) Laboratory, George Mason University, Manassas, VA, United States; ^3^School Life and Health Sciences, University of Roehampton, London, United Kingdom; ^4^Haute Ecole Bruxelles-Brabant (HE2B-ISEK), Brussels, Belgium

**Keywords:** fatigue, energy, vigor, moods, biomarkers

Fatigue is a complex and often misunderstood mood. Most biomedical researchers interested in fatigue conceptually defining fatigue as the absence of energy. However, Loy et al. (2018) provided evidence that biologically energy and fatigue may indeed be two distinct unipolar moods. Since then, multiple studies have provided evidence that energy and fatigue may have both distinct and overlapping biological (Loy and O'Connor, 2016; Eshragh et al., 2017; Boolani et al., 2019a, 2022a; Dupree et al., 2019), behavioral (Boolani and Manierre, 2019; Boolani et al., 2019a; Papadakis et al., 2022), and biomechanical correlates (Boolani et al., 2020, 2022a; Kowalski et al., 2021; Mahoney et al., 2021; Sprague et al., 2021; Kadry et al., 2022). The strength of this evidence is mixed with several studies providing evidence from randomized controlled crossover designs (Loy and O'Connor, 2016; Dupree et al., 2019; Boolani et al., 2020), while others relied on cross-sectional designs (Eshragh et al., 2017; Boolani and Manierre, 2019; Boolani et al., 2019b, 2022b; Kowalski et al., 2021; Mahoney et al., 2021; Kadry et al., 2022; Papadakis et al., 2022) and two studies examined longitudinal data (Sprague et al., 2021; Boolani et al., 2022c), while another was a review of literature (Loy et al., 2018). Taken together these studies provide compelling arguments for measuring energy and fatigue as two distinct moods.

The case for measuring both energy and fatigue is most effectively illustrated by Sprague et al. (2021) and Zhang et al., who provided evidence that declines in feelings of energy and increase in feelings of fatigue increase risk of mortality. Sprague et al. (2021) provided evidence that declines in feelings of energy significantly increased risks for mortality and decreased functional capacity, while changes in feelings of fatigue did not. Conversely Zhang et al. report that a combination of frailty, a measure consisting of fatigue, and neutrophil-to-lymphocyte ratio (NLR), a marker of fatigue (Loy et al., 2018), had the greatest impact on mortality in patients with a diagnosis of cancer (Zhang et al.). These studies (Zhang et al.; Sprague et al., 2021) provide conflicting information in terms of the role that energy and fatigue play in mortality. However, these opposing findings may be explained by the fact that Sprague et al. (2021) measured energy and fatigue as two distinct constructs, in what started as a healthy population, whereas Zhang et al. addressed a population of cancer patients. Prior evidence supports that patients with cancer can have either low energy or high fatigue or both (Eshragh et al., 2017). The methodological differences in the measurement of fatigue and energy, as well as using different populations, can explain discrepancies of these two studies (Zhang et al.; Sprague et al., 2021).

In addition to the evidence that changes in prolonged feelings of energy impact mobility (Sprague et al., 2021), Boolani et al. (2020) provided convincing data that declines in current feelings of mental energy led to significant declines in balance control in older adults, since many of their participants presented clinically relevant declines on the Berg Balance Test. These findings (Boolani et al., 2020) suggest that one must account for current feelings of energy when performing physical tasks. To that end, Keegan et al. sought to develop an Acute Readiness Monitoring Scale (ARMS) to assess both energy and fatigue in order to identify readiness of military personnel. This 32-item tool is easy to administer, has six subscales and assesses a combination of mental and physical energy and fatigue (Keegan et al.). Summers et al. further validated this scale by testing its sensitivity and found that scores on all six subscales of the ARMS changed in response to sleep deprivation. Together these findings provided a novel, low-cost tool that can be used to assess both mental and physical energy and fatigue (Keegan et al.; Summers et al.) in military personnel.

While Keegan et al. and Summers et al. argued for the need to measure mental and physical energy and fatigue, Filippi et al. provided the most compelling evidence to further deconstruct energy and fatigue into motor, cognitive, social, emotional, and sexual. To further specify, they also devided the state as prolonged or aiming at trait aspects of these sensations. Using previously published evidence, Fillippi et al. reviewed literature that found objective and subjective parameters of state, prolonged state and trait fatigue are associated with core body temperature, the HPA axis, glucose metabolism, mitochondrial function, the autonomic nervous system and inflammation; however, they also suggest that these biological correlates may be associated with state, prolonged state and trait levels energy based on emerging evidence. These findings (Filippi et al.) support the need to measure trait, prolonged state and state level energy and fatigue going forward.

Recently several studies have identified how trait level energy and fatigue modify state level energy and fatigue in response to various interventions (Manierre et al., 2020; Fuller et al., 2021; Boolani et al., 2022a). The idea of trait level features influencing state level energy and fatigue was recently highlighted by a topical review (Behm and Carter) that suggested empathy might increase feelings of fatigue during exercise. Behm and Carter caveat their working hypothesis by suggesting that sex, age, cultural, exercise type and personality differences may modify the effects of empathy on fatigue. The authors combined the mirror neuron and affordance competition hypotheses to generate their working hypothesis on the role of empathy increasing feelings of fatigue (Behm and Carter). While both hypotheses support how empathy may increase feelings of fatigue, the affordance competition hypothesis suggests that cognitive demands during exercise may enhance negative mood affect, a dual task paradigm used by researchers in the past to increase mental fatigue (Greig et al., 2007).

Interestingly, Meixner and Herbert used this dual-task paradigm to identify whether attentional focus during exercise influences psychophysiological responses to an acute bout of exercise, including positive and negative affect, which have previously been associated with feelings of energy and fatigue, respectively (Watson et al., 1988). Although these authors found that attentional focus did not negatively impact psychophysiological responses to an acute bout of exercise, they report the benefits of an acute bout of exercise on both positive and negative affect (Meixner and Herbert). These findings are in line with a previously published meta-analysis which concluded that a single bout of exercise increased feelings of energy and depending on intensity and time may also reduce feelings of fatigue (Loy et al., 2013). While, Meixner and Hebert used a 15-min bout of exercise to enhance affect, Carmichael et al. tried to enhance feelings of energy and fatigue using a 4-min bout of stair climbing. Carmichael et al. reported that while 4 min of stair walking significantly increased heart rate and RPE, there were no significant differences in feelings of energy and fatigue after this short bout of physical activity. Previously, Boolani et al. (2019a) had reported that 6 min of self-selected walking speed increased feelings of energy and decreased feelings of fatigue (Boolani et al., 2019a). Taken together these studies (Carmichael et al.; Meixner and Hebert; Loy et al., 2013; Boolani et al., 2019a) suggest that, an acute bout of exercise will positively impact affect moods, but a minimum of 6 min of physical activity is needed to positively enhance moods.

Though significant evidence exists on the beneficial effects of an acute bout of exercise on moods (Carmichael et al.; Meixner and Hebert; Loy et al., 2013; Boolani et al., 2019a), until recently, the evidence on the effects of chronic exercise on feelings of energy and fatigue was mixed and suggested the influence of placebo effects on changes in energy and fatigue (O'Connor and Puetz, 2005; Puetz et al., 2006). In 2022 Wender et al. published a meta-analysis of 81 studies (7,050 participants) that considered potential moderating variables in their analyses. This meta-analysis reported that chronic exercise has a small, but significant effect on decreasing feelings of fatigue, a small-to-moderate effect on increasing feelings of energy and a moderate effect on increasing feelings of vitality (Wender et al.). Their analyses suggested that several factors such as exercise intensity and duration influenced feelings of fatigue, exercise intensity and modality influenced feelings of energy and participant health, exercise intensity, modality, and exercise training location influenced feelings of vitality (Wender et al.). Taken together, these findings suggest that exercise can positively influence feelings of energy and fatigue both after an acute (Carmichael et al.; Meixner and Hebert; Loy et al., 2013; Boolani et al., 2019a) bout and with chronic exercise (Wender et al.; O'Connor and Puetz, 2005; Puetz et al., 2006).

In summary, current evidence suggests that energy and fatigue should be measured as two distinct moods, and that one must account for trait, prolonged state and state energy and fatigue, while also accounting for cognitive, physical, social, emotional, and sexual aspects of these moods (Filippi et al.). Based on the evidence, trait and prolonged state energy may increase mortality rates in healthy older adults (Sprague et al., 2021), while increased prolonged state fatigue may increase mortality rates in patients with cancer (Zhang et al.). However, acute declines in state energy may also have detrimental effects (Boolani et al., 2020), and for tactical athletes these effects may be fatal. The ARMS survey is a valid and reliable low-cost way to assess both mental and physical energy and fatigue (Keegan et al.; Summers et al.) in these tactical athletes. Exercise may be an intervention that can be used to enhance feelings of energy and reduce feelings of fatigue both in an acute (Carmichael et al.; Meixner and Hebert; Loy et al., 2013; Boolani et al., 2019a) and chronic (Wender et al.; O'Connor and Puetz, 2005; Puetz et al., 2006) settings, and changes in acute attention focus do not temper the positive influence of exercise on moods (Meixner and Hebert). However, a working hypothesis on being empathic toward others during exercise dampening the potential anti-fatiguing benefits of exercise (Behm and Carter) should be explored further. Based on current evidence future researchers should further explore optimal exercise level and frequency to improve feelings of energy and fatigue considering individuals abilities.

## Author contributions

AB wrote the initial draft of the editorial. All authors read and edited the final manuscript. All authors contributed to the article and approved the submitted version.
